# Two New Diphenylketones and a New Xanthone from *Talaromyces islandicus* EN-501, an Endophytic Fungus Derived from the Marine Red Alga *Laurencia okamurai*

**DOI:** 10.3390/md14120223

**Published:** 2016-12-07

**Authors:** Hong-Lei Li, Xiao-Ming Li, Hui Liu, Ling-Hong Meng, Bin-Gui Wang

**Affiliations:** 1 Laboratory of Marine Biology and Biotechnology, Qingdao National Laboratory for Marine Science and Technology, Key Laboratory of Experimental Marine Biology, Institute of Oceanology, Chinese Academy of Sciences, Nanhai Road 7, Qingdao 266071, China; lihonglei428@126.com (H.-L.L.); lixmqdio@126.com (X.-M.L.); liuhui625@163.com (H.L.); 2 University of Chinese Academy of Sciences, Yuquan Road 19A, Beijing 100049, China

**Keywords:** *Laurencia okamurai*, *Talaromyces islandicus*, diphenylketone, xanthone, antioxidative activity, antibacterial activity

## Abstract

Two new diphenylketones (**1** and **2**), a new xanthone (**3**), and a known xanthone analogue (**4**) were isolated and identified from *Talaromyces islandicus* EN-501, an endophytic fungus obtained from the fresh collected marine red alga *Laurencia okamurai*. Their structures were elucidated on the basis of NMR spectroscopic and X-ray crystallographic analysis. The joint isolation of benzophenones and xanthones from the same fungal strain supports the biogenesis of xanthones via a benzophenone intermediate. It is worth mentioning that xanthones **3** and **4** have a methyl group at C-6 and C-2, respectively, which is uncommon compared with typical xanthones usually having a methyl group at C-8. Compounds **1**–**4** exhibited potent antioxidative activities against DPPH (1,1-diphenyl-2-picrylhydrazyl) and ABTS (2,2′-azino-bis(3-ethylbenzothiazoline-6-sulphonate) radicals with IC_50_ values ranging from 0.58 to 6.92 μg/mL, which are stronger than that of the positive controls BHT (butylated hydroxytoluene) and ascorbic acid. Compounds **1**, **3**, and **4** also showed inhibitory activities against several pathogenic bacteria.

## 1. Introduction

**Figure 1:**
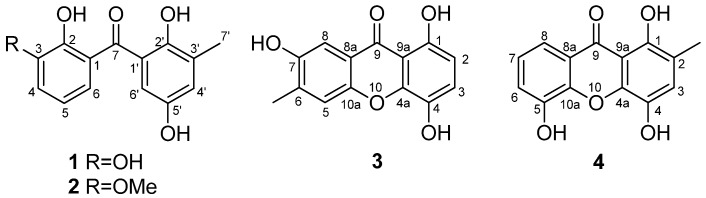
Structures of the isolated compounds **1**–**4**.

Marine-derived fungi have been evidenced as a prolific source for the discovery of pharmacologically-active natural products [1,2]. As part of our ongoing research toward the discovery of secondary metabolites from marine-derived fungi [3,4,5,6,7,8], an endophytic fungal strain, *Talaromyces islandicus* EN-501, was selected for chemical investigation. Recently, several new natural products were isolated from plant-derived endophytic species of the genus *Talaromyces* [9,10,11]. The fungal strain used in the present work was isolated from the inner tissue of the fresh collected marine red alga *Laurencia okamurai*. As a result, two new diphenylketones (**1** and **2**), a new xanthone derivative (**3**), and a known xanthone analogue (**4**) (Figure 1), were isolated and identified. It should be mentioned that compound **4** was listed as a substance tested for antitermitic activity [12], but no spectroscopic data or source information were reported. The structure elucidation and fully assigned NMR data of **4** are first described herein. The antioxidative activities against DPPH (1,1-diphenyl-2-picrylhydrazyl) and ABTS (2,2′-azino-bis(3-ethylbenzothiazoline-6-sulphonate) radicals, as well as antibacterial activity against six pathogenic strains, were evaluated. This paper describes the isolation, characterization, and bioactivity of compounds **1**–**4**.

## 2. Results and Discussion

### 2.1. Structure Elucidation of the New Compounds

**Figure 2:**
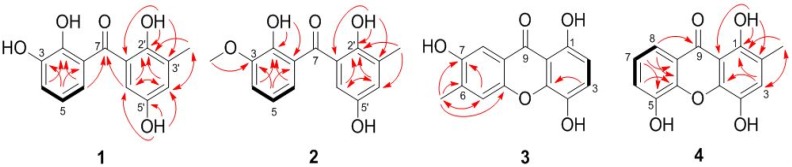
Key ^1^H-^1^H COSY (bold lines) and HMBC (red arrows) correlations of compounds **1**–**4**.

**Figure 3:**
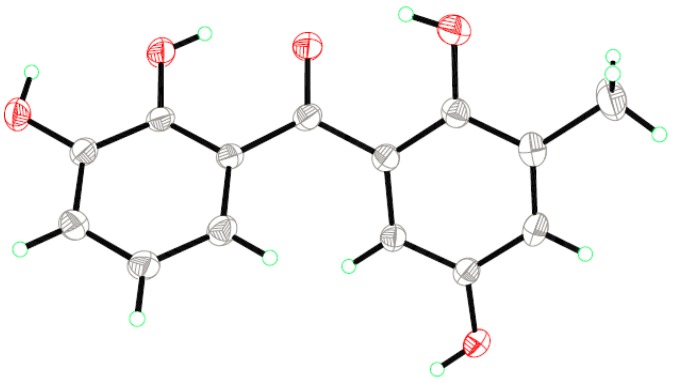
X-ray structure of compound **1**.

Compound **1** was obtained as yellowish crystals with the molecular formula C_14_H_12_O_5_ as established by HRESIMS (high resolution electrospray ionization mass spectroscopy, Figure S1) data, indicating nine degrees of unsaturation. The ^1^H NMR spectrum (Figure S2) showed signals for a 1,2,3-trisubstituted phenyl group at Δ_H_ 6.98 (d, *J* = 7.7 Hz, H-4), 6.77 (t, *J* = 7.7, Hz, H-5), and 6.71 (d, *J* = 7.6 Hz, H-6), and a 1,2,3,5-tetrasubstituted phenyl unit at Δ_H_ 6.91 (br s, H-4′) and 6.62 (br s, H-6′) (Table 1). The ^13^C NMR spectrum (Figure S3) displayed 14 resonances, which were classified by DEPT (distortionless enhancement by polarization transfer, Figure S3) experiments as one methyl, five aromatic methines, and eight non-protonated (including one ketone and seven aromatic) carbons (Table 1). The ^1^H and ^13^C NMR spectroscopic data (Figures S2–S6) are evocative of a benzophenone scaffold [13]. In the HMBC spectrum (Figure S6), the correlations from the proton of 2′-OH (Δ_H_ 11.42) to C-1′, C-2′, and C-3′ located the OH group at C-2′, while the correlations from H-7′ (Δ_H_ 2.18) to C-2′, C-3′, and C-4′ placed the methyl group at C-3′, and at last, the correlations from the OH proton at Δ_H_ 9.02 to C-4′, C-5′, and C-6′ determined this OH group at C-5′. As for the other nucleus, HMBC correlations from H-6 to C-2 and C-7, from H-5 to C-1 and C-3, and from H-4 to C-2, as well as the chemical shifts of C-2 and C-3 at Δ_C_ 143.8 and 145.8 determined the locations of two other phenolic OH groups at C-2 and C-3 (Figure 2). Thus, compound **1** was characterized as 2,2′,3,5′-tetrahydroxy-3′-methylbenzophenone, and the structure was confirmed by the single-crystal X-ray diffraction analysis (Figure 3).

Compound **2** was obtained as a yellowish solid with the molecular formula C_15_H_14_O_5_, as established by HRESIMS data (Figure S7), indicating nine degrees of unsaturation. The 1D and 2D NMR spectra data of **2** (Table 1, Figures S8–S12) are reminiscent of those of **1**, only differing in the additional resonances from a methoxy group at Δ_H_ 3.93 and Δ_C_ 56.3 in the NMR spectra of **2**. In the HMBC spectrum, correlation from the protons of the OMe group to C-3 assigned the methoxy group at C-3 (Figure 2). Therefore, the structure of compound **2** was identified as 2,2′,5′-trihydroxy-3-methoxy-3′-methylbenzophenone.

Compound **3** was also obtained as a yellowish solid with the molecular formula C_14_H_10_O_5_, as established by HRESIMS data (Figure S13), indicating ten degrees of unsaturation. Extensive analysis of the ^1^H and ^13^C NMR data (Table 2, Figures S14 and S15) as well as the COSY and HSQC (heteronculear single quantum coherence) spectra (Figures S16 and S17) disclosed that **3** was a xanthone analogue similar to 1,4,7-trihydroxy-xanthone [14]. The main differences are that resonance for one of the five aromatic protons in the ^1^H NMR spectrum of 1,4,7-trihydroxy-xanthone disappeared in that of **3**, and signal for an additional methyl group was observed at Δ_H_ 2.19 and Δ_C_ 14.5 (CH_3_-6) in the NMR spectra of **3**. Moreover, the resonance of C-6—compared to that of the known xanthone—moved to the shielded field in the ^13^C NMR spectrum of **3**. These spectroscopic features suggested that compound **3** was 1,4,7-trihydroxy-6-methylxanthone. The COSY and HMBC correlations (Figure 2, Figures S16 and S18) supported the above deduction.

Compound **4** was obtained as a yellowish solid with the molecular formula C_14_H_10_O_5_, the same as that of **3**, as established by HRESIMS data (Figure S19). The ^1^H and ^13^C NMR data of **4** (Table 2, Figures S20 and S21) as well as the COSY and HSQC spectra (Figures S22 and S23) showed a close relationship to that of **3**. The methylation of C-2 and hydroxylation of C-5 in **4** were supported by the HMBC correlations from CH_3_-2 to C-1, C-2, and C-3 and from H-7 to C-5, respectively (Figure 2 and Figure S24). Thus, compound **4** was characterized as 1,4,5-trihydroxy-2-methylxanthone. This compound was listed as a substance tested for antitermitic activity [12], but no spectroscopic data or source information were reported. So, its structure elucidation and fully assigned NMR data are provided here.

The biogenesis for most xanthones is known to proceed via a benzophenone intermediate in lichens and fungi [15,16]. The joint isolation of benzophenones and xanthones from *T. islandicus* EN-501 indicates that benzophenone (**1**) might serve as an intermediate to afford xanthone (**4**) upon cyclization. Compound **4** is an atypical xanthone with a methyl group at C-2, which is different from most other xanthones that usually possess a methyl group either at C-3/C-6 or at C-1/C-8 [15,16]. Atypical xanthones with a methyl at C-2/C-7 have been described previously [17,18], which might be derived from a site-selective methylation step involving discrete enzymes [17].

### 2.2. Biological Activities of the Isolated Compounds

In the antioxidative assay, compounds **1**–**4** exhibited comparable DPPH radical scavenging activity, with IC_50_ values ranging from 1.23 to 6.92 μg/mL (Table 3), stronger than that of BHT (butylated hydroxytoluene), a well-known antioxidant (IC_50_ = 16.27 μg/mL). In addition, compounds **1**–**4** showed potent ABTS radical scavenging activity with IC_50_ values ranging from 0.58 to 2.35 μg/mL (Table 3), which are stronger than that of ascorbic acid (IC_50_ = 3.01 μg/mL). Compounds **1**–**4** were also evaluated for their antibacterial activities. While compounds **1**, **3**, and **4** showed potent activities against three human pathogens (*Escherichia coli*, *Pseudomonas aeruginosa*, and *Staphylococcus aureus*) and three aquatic bacteria (*Vibrio alginolyticus*, *V. harveyi*, and *V. parahaemolyticus*), with minimum inhibitory concentration (MIC) values ranging from 4 to 32 μg/mL (Table 4), compound **2** showed weak activity against the tested bacteria (IC_50_ > 64 μg/mL), suggesting that methoxylation at C-3 weakened the antibacterial activities (**1** vs. **2**).

## 3. Experimental Section

### 3.1. General

Melting points were determined with an SGW X-4 micro-melting-point apparatus. UV spectra were measured using a Lengguang Gold S54 spectrophotometer (Shanghai Lengguang Technology Co.Ltd., Shanghai, China). NMR spectra were acquired using a Bruker Avance 500 spectrometer (Bruker Biospin Group, Karlsruhe, Germany). Mass spectra were measured on a VG Autospec 3000 mass spectrometer (VG Instruments, London, UK) or an API QSTAR Pulsar 1 mass spectrometer (Applied Biosystems, Foster City, CA, USA). HPLC analysis was carried out on a Dionex HPLC system (P680 HPLC pump, UVD 340U UV-visible detector) using a C_18_ column (5 μm, 8.0 mm i.d. × 250 mm). Column chromatography (CC) was performed with Si gel (200–300 mesh, Qingdao Haiyang Chemical Factory, Qingdao, China), Lobar LiChroprep RP-18 (40–60 μm, Merck, Darmstadt, Germany), and Sephadex LH-20 (18–110 μm, Merck).

### 3.2. Fungal Material

The fungal endophyte, *Talaromyces islandicus* EN-501, was isolated following surface sterilization from the marine red alga *Laurencia okamurai* collected from the coast of Qingdao, China, by using the procedures provided in our earlier report [19]. The isolate was identified by analysis of its morphological characteristics and ITS (internal transcribed spacer) gene sequence, which has been submitted to GenBank (accession number KU885935). A BLAST search result indicated that the sequence is almost same (99%) to the sequence of *Talaromyces islandicus* CBS 117284 (compared with KF984882.1). The strain was preserved in the Institute of Oceanology, Chinese Academy of Sciences (IOCAS).

### 3.3. Fermentation

The fermentation was carried out statically in solid rice medium in 1 L Erlenmeyer flasks (each containing 70 g rice, 0.1 g corn flour, 0.3 g peptone, 0.1 g sodium glutamate, and 100 mL naturally sourced and filtered seawater, which was obtained from the Huiquan Gulf of the Yellow Sea near the campus of IOCAS, pH 6.5–7.0) for 30 days at room temperature.

### 3.4. Extraction and Isolation

The fermented whole culture (90 flasks) was extracted with EtOAc to afford an organic extract (80 g), which was partitioned by Si gel vacuum liquid chromatography (VLC) using different solvents of increasing polarity, from petroleum ether (PE) to methanol (MeOH) to yield nine fractions (Frs. 1–9) based on thin layer chromatography (TLC) and HPLC analysis. Fr.4 (5.5 g), eluted with PE–EtOAc (2:1), was further chromatographed over reversed-phase C_18_ eluting with a MeOH–H_2_O gradient (from 1:9 to 1:0, *v*/*v*) to afford seven subfractions (Fr.4.1–Fr.4.7). Fr.4.4 (0.5 g) was purified by CC on Si gel (CHCl_3_–MeOH, from 100:1 to 20:1, *v*/*v*) and then on Sephadex LH-20 (MeOH) to yield compound **1** (120.0 mg), while Fr.4.5 (0.4 g) was purified by CC on Si gel (CHCl_3_–MeOH, from 80:1 to 20:1, *v*/*v*) and then on Sephadex LH-20 (MeOH) to yield compound **2** (5.3 mg). Fr.4.6 (0.6 g) was purified by CC on Si gel (CHCl_3_–acetone, from 40:1 to 3:1, *v*/*v*) and Sephadex LH-20 (MeOH), and then purified by prep. TLC (plate: 20 × 20 cm, developing solvents: PE/EtOAc, 2:1) to yield compounds **3** (8.0 mg) and **4** (10.2 mg).

*2,2′,3,5-Tetrahydroxy-3′-methylbenzophenone* (**1**): Yellowish crystals; mp 174–176 °C; UV (MeOH) λ_max_ (log ε) 203 (4.56), 208 (4.58), 221 (4.75), 268 (4.56), 376 (4.16) nm; ^1^H and ^13^C NMR data, see Table 1. HRESIMS *m*/*z* 261.0755 ([M + H]^+^) (calcd. for C_14_H_13_O_5_, 261.0757, Δ 0.2 ppm).

*2,2′,5′-Trihydroxy-3-methoxy-3′-methylbenzophenone* (**2**): Yellowish solid; UV (MeOH) λ_max_ (log ε) 203 (4.25), 209 (4.33), 226 (4.42), 266 (4.26), 383 (3.82) nm; ^1^H and ^13^C NMR data, see Table 1. HRESIMS *m*/*z* 275.0912 ([M + H]^+^) (calcd. for C_15_H_15_O_5_, 275.0914, Δ 0.2 ppm).

*1,4,7-Trihydroxy-6-methylxanthone* (**3**): Yellowish solid; UV (MeOH) λ_max_ (log ε) 203 (4.92), 213 (4.99), 248 (4.96), 259 (4.92), 314 (4.59) nm; ^1^H and ^13^C NMR data, see Table 2. HRESIMS *m*/*z* 259.0599 ([M + H]^+^) (calcd. for C_14_H_11_O_5_, 259.0601, Δ 0.2 ppm).

*1,4,5-Trihydroxy-2-methylxanthone* (**4**): Yellowish solid; UV (MeOH) λ_max_ (log ε) 205 (4.86), 212 (4.96), 248 (4.99), 263 (4.97), 317 (4.52) nm; ^1^H and ^13^C NMR data, see Table 2. HRESIMS *m*/*z* 259.0598 ([M + H]^+^) (calcd. for C_14_H_11_O_5_, 259.0601, Δ 0.3 ppm).

### 3.5. X-ray Crystallographic Analysis of Compound ***1*** [20]

The crystallographic data were collected on a Bruker Smart-1000 CCD diffractometer equipped with a graphite-monochromatic Mo-*K*α radiation (λ = 0.71073 Å) at 298(2) K. The data were corrected for absorption by using the program SADABS [21]. The structure was solved by direct methods with the SHELXTL software package [22]. All non-hydrogen atoms were refined anisotropically. The H atoms were located by geometrical calculations, and their positions and thermal parameters were fixed during the structure refinement. The structure was refined by full-matrix least-squares techniques [23].

*Crystal data for compound* **1**: C_14_H_12_O_5_, F.W. = 260.24, orthorhombic space group Pna2(1), unit cell dimensions *a* = 8.1951(7) Å, *b* = 17.9481(15) Å, *c* = 8.1962(8) Å, *V* = 1205.55(19) Å^3^, α = γ = β = 90°, *Z* = 4, *d*_calcd_ = 1.434 mg/m^3^, crystal dimensions 0.42 × 0.40 × 0.37 mm, μ = 0.110 mm^−1^, *F*(000) = 544. The 5770 measurements yielded 2070 independent reflections after equivalent data were averaged, and Lorentz and polarization corrections were applied. The final refinement gave *R*_1_ = 0.0383 and w*R*_2_ = 0.0789 (*I* > 2σ(*I*)).

### 3.6. Antioxidant Assay

Evaluation of pure compounds for antioxidative activity against DPPH and ABTS free radicals was carried out by the method described previously [8]. BHT and ascorbic acid were used as positive controls against DPPH and ABTS free radicals, respectively.

### 3.7. Antimicrobial Assay

Antimicrobial evaluation against three human pathogens (*E. coli*, *P. aeruginosa*, *S. aureus*) and three aquatic bacteria (*V. alginolyticus*, *V. harveyi*, and *V. parahaemolyticus*) was carried out by the microplate assay [24]. Chloramphenicol was used as positive control.

## 4. Conclusions

In summary, two new diphenylketones (**1** and **2**), a new xanthone (**3**), and a known xanthone analogue (**4**) were isolated and identified from the marine algal-derived endophytic fungus *T. islandicus* EN-501. Each of these compounds exhibited potent antioxidative activities against DPPH and ABTS radicals, and compounds **1**, **3**, and **4** showed strong antibacterial activities.

## Figures and Tables

**Table 1:** ^1^H and ^13^C NMR data of compounds **1** and **2** (Δ in ppm, *J* in Hz).

Position	1 (Measured in DMSO-*d*_6_)		2 (Measured in CDCl_3_)	
	Δ_C_	Δ_H_	Δ_C_	Δ_H_
1	126.1, C		122.1, C	
2	143.8, C		148.5, C	
3	145.8, C		148.2, C	
4	117.7, CH	6.98, d (7.7)	123.1, CH	7.11, d (7.0)
5	119.2, CH	6.77, t (7.7)	118.9, CH	6.89, t (7.2)
6	119.0, CH	6.71, d (7.6)	115.2, CH	7.04, d (7.0)
7	202.5, C		201.7, C	
1′	120.2, C		119.0, C	
2′	152.3, C		155.0, C	
3′	127.1, C		129.0, C	
4′	125.3, CH	6.91, br s	125.6, CH	6.94, br s
5′	148.7, C		146.5, C	
6′	114.8, CH	6.62, br s	115.5, CH	6.84, br s
7′	15.4, CH_3_	2.18, s	15.8, CH_3_	2.27, s
2-OH				9.00, s
3-OH/OMe		9.47, s	56.3, CH_3_	3.93, s
2′-OH		11.42, s		10.95, s
5′-OH		9.02, s		4.57, s

**Table 2:** ^1^H and ^13^C NMR data of compounds **3** and **4** (Δ in ppm, *J* in Hz).

Position	3 (Measured in DMSO-*d*_6_)		4 (Measured in DMSO-*d*_6_)	
	Δ_C_	Δ_H_	Δ_C_	Δ_H_
1	152.0, C		149.7, C	
2	110.6, CH	7.42, d (7.2)	117.6, C	
3	124.5, CH	7.24, d (7.2)	124.6, CH	7.21, s
4	137.9, C		137.0, C	
4a	146.2, C		141.0, C	
5	123.8, CH	7.20, s	147.6, C	
6	117.1, C		120.8, CH	7.32, d (7.6)
7	149.1, C		124.3, CH	7.27, t (7.8)
8	120.9, CH	7.26, s	113.8, CH	7.54, d (7.7)
8a	120.5, C		120.6, C	
9	182.8, C		182.3, C	
9a	107.7, C		107.8, C	
10a	141.2, C		145.1, C	
2-CH_3_			14.4, CH_3_	2.17, s
6-CH_3_	14.5, CH_3_	2.19, s		
1-OH		12.04, s		12.06, s

**Table 3:** Antioxidant activity of compounds **1**–**4** against 1,1-diphenyl-2-picrylhydrazyl (DPPH) and 2,2′-azino-bis(3-ethylbenzothiazoline-6-sulphonate (ABTS) (IC_50_, μg/mL).

Samples	1	2	3	4	BHT ^a^	Ascorbic Acid
DPPH	1.26	1.33	6.92	1.23	16.27	
ABTS	0.69	0.58	2.35	1.27		3.01

^a^ BHT: butylated hydroxytoluene.

**Table 4:** Antibacterial activity of compounds **1**–**4** (minimum inhibitory concentration, MIC, μg/mL) ^a^.

Samples	1	2	3	4	Chloramphenicol
EC	4	>64	32	4	1
PA	4	>64	32	4	4
SA	8	>64	>64	8	2
VA	4	>64	32	4	0.5
VH	8	>64	32	8	2
VP	4	>64	32	4	2

^a^ EC: *E*. *coli*; PA: *P*. *aeruginosa*; SA: *S*. *aureus*; VA: *V*. *alginolyticus*; VH: *V*. *harveyi*; VP: *V*. *parahaemolyticus*.

## Supplementary Materials

The following are available online at www.mdpi.com/1660-3397/14/12/223/s1, Figure S1: HRESIMS spectrum of compound **1**, Figure S2: ^1^H NMR (500 MHz, DMSO-*d*_6_) of compound **1**, Figure S3: ^13^C NMR and DEPT (125 MHz, DMSO-*d*_6_) of compound **1**, Figure S4: COSY spectrum of compound **1**, Figure S5: HSQC spectrum of compound **1**, Figure S6: HMBC spectrum of compound **1**, Figure S7: HRESIMS spectrum of compound **2**, Figure S8: ^1^H NMR (500 MHz, CDCl_3_ ) of compound **2**, Figure S9: ^13^C NMR and DEPT (125 MHz, CDCl_3_) of compound **2**, Figure S10: COSY spectrum of compound **2**, Figure S11: HSQC spectrum of compound **2**, Figure S12: HMBC spectrum of compound **2**, Figure S13: HRESIMS spectrum of compound **3**, Figure S14: ^1^H NMR (500 MHz, DMSO-*d*_6_) of compound **3**, Figure S15: ^13^C NMR and DEPT (125 MHz, DMSO-*d*_6_) of compound **3**, Figure S16: COSY spectrum of compound **3**, Figure S17: HSQC spectrum of compound **3**, Figure S18: HMBC spectrum of compound **3**, Figure S19: HRESIMS spectrum of compound **4**, Figure S20: ^1^H NMR (500 MHz, DMSO-*d*_6_) of compound **4**, Figure S21: ^13^C NMR and DEPT (125 MHz, DMSO-*d*_6_) of compound **4**, Figure S22: COSY spectrum of compound **4**, Figure S23: HSQC spectrum of compound **4**, Figure S24: HMBC spectrum of compound **4**.
