# Supplementary material for: Two New Diphenylketones and a New Xanthone from Talaromyces islandicus EN-501, an Endophytic Fungus Derived from the Marine Red Alga Laurencia okamurai
Source: Mar Drugs. 2016 Dec 7;14(12):223. doi: 10.3390/md14120223 (PMC5192460; doi:10.3390/md14120223)
Supplement: Supplementary file 1 [file marinedrugs-14-00223-s001.docx]

SupplementaryMaterials: Two New Diphenylketones and a New Xanthone from *Talaromyces islandicus* EN-501, an Endophytic Fungus Derived from the Marine Red Alga *Laurencia okamurai*

Hong-Lei Li, Xiao-Ming Li, Hui Liu, Ling-Hong Meng and Bin-Gui Wang


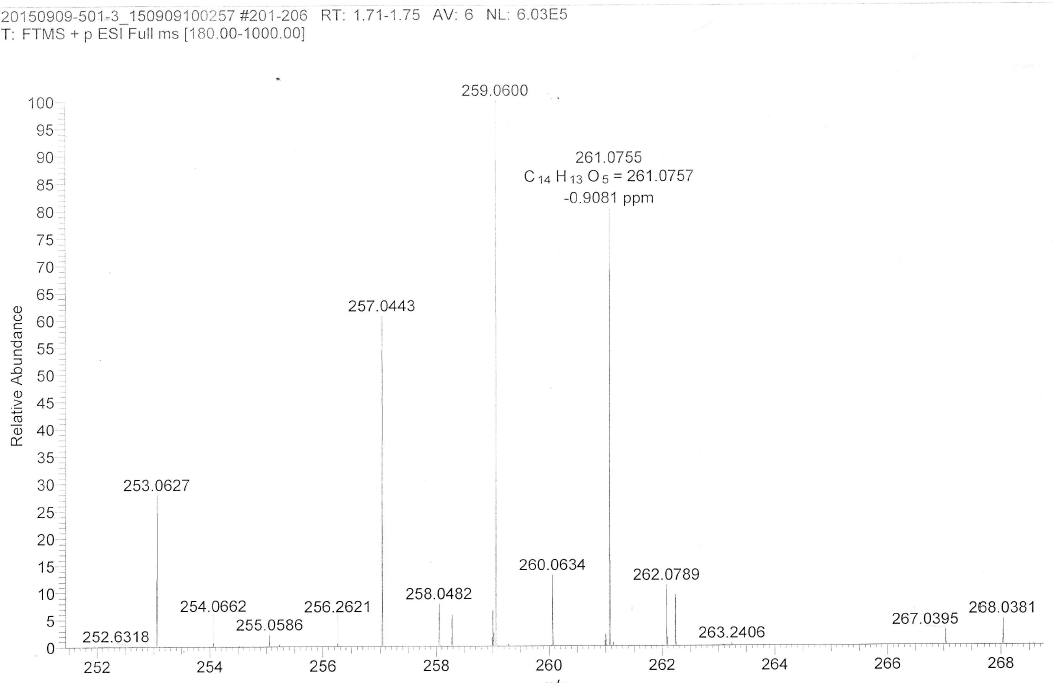


**Figure S1.** HRESIMS spectrum of compound **1**.


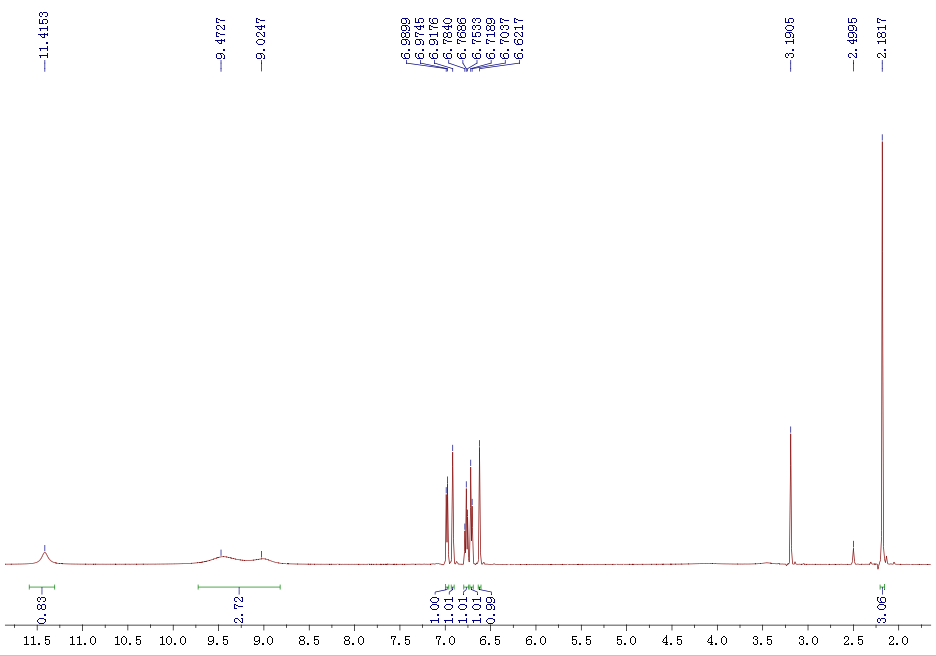


**Figure S2.**^1^H NMR (500 MHz, DMSO-*d*_6_) of compound **1**.


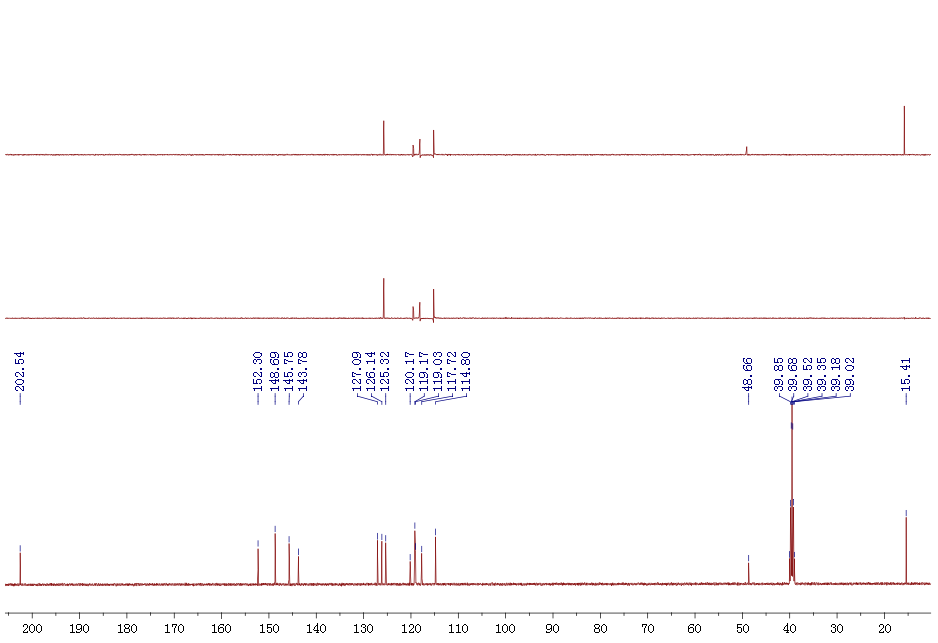


**Figure S3.**^13^C NMR and DEPT (125 MHz, DMSO-*d*_6_) of compound **1**.


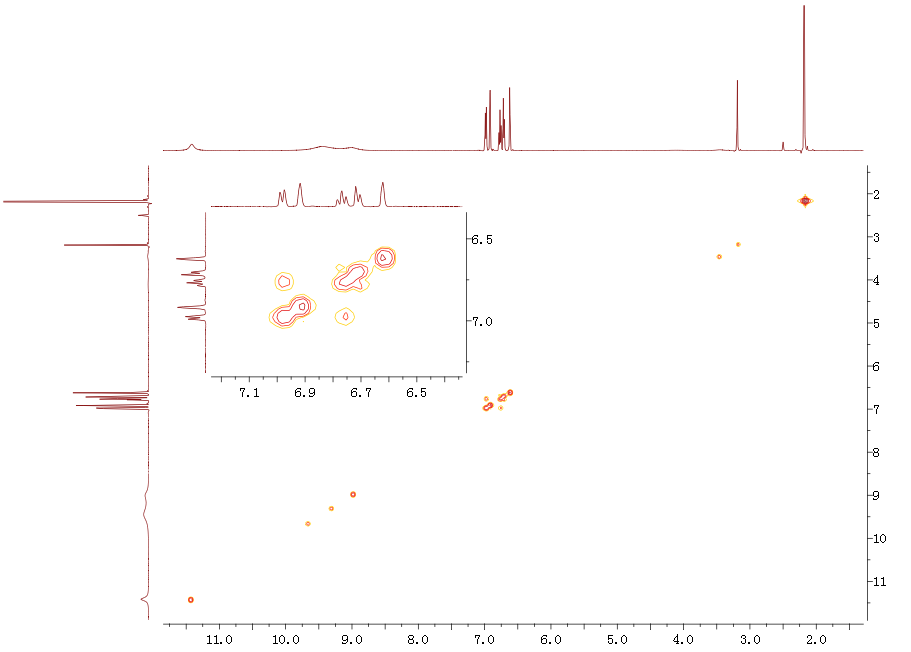


**Figure S4.** COSY spectrum of compound **1**.


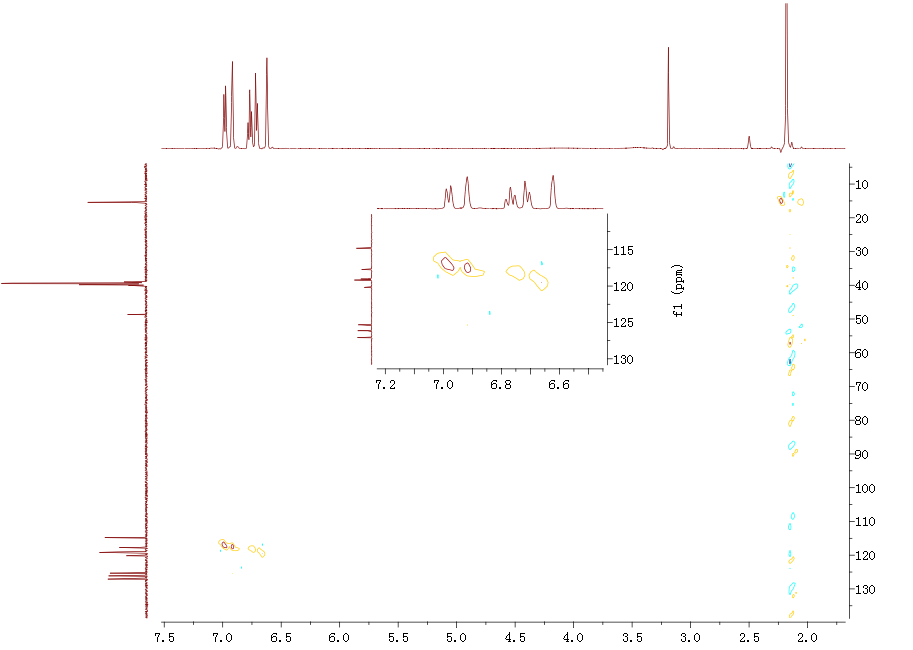


**Figure S5.** HSQC spectrum of compound **1**.


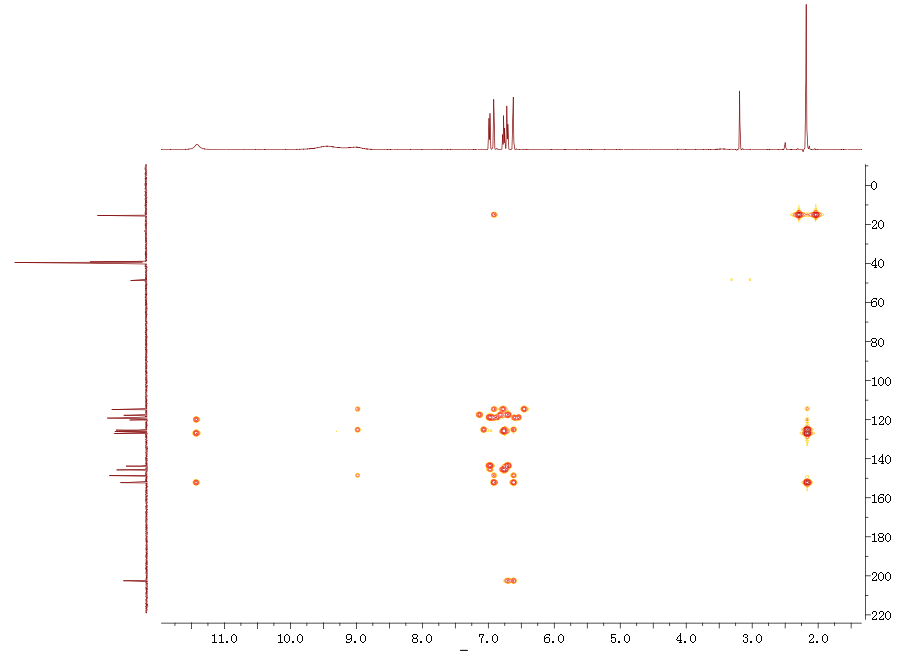


**Figure S6.** HMBC spectrum of compound **1**.


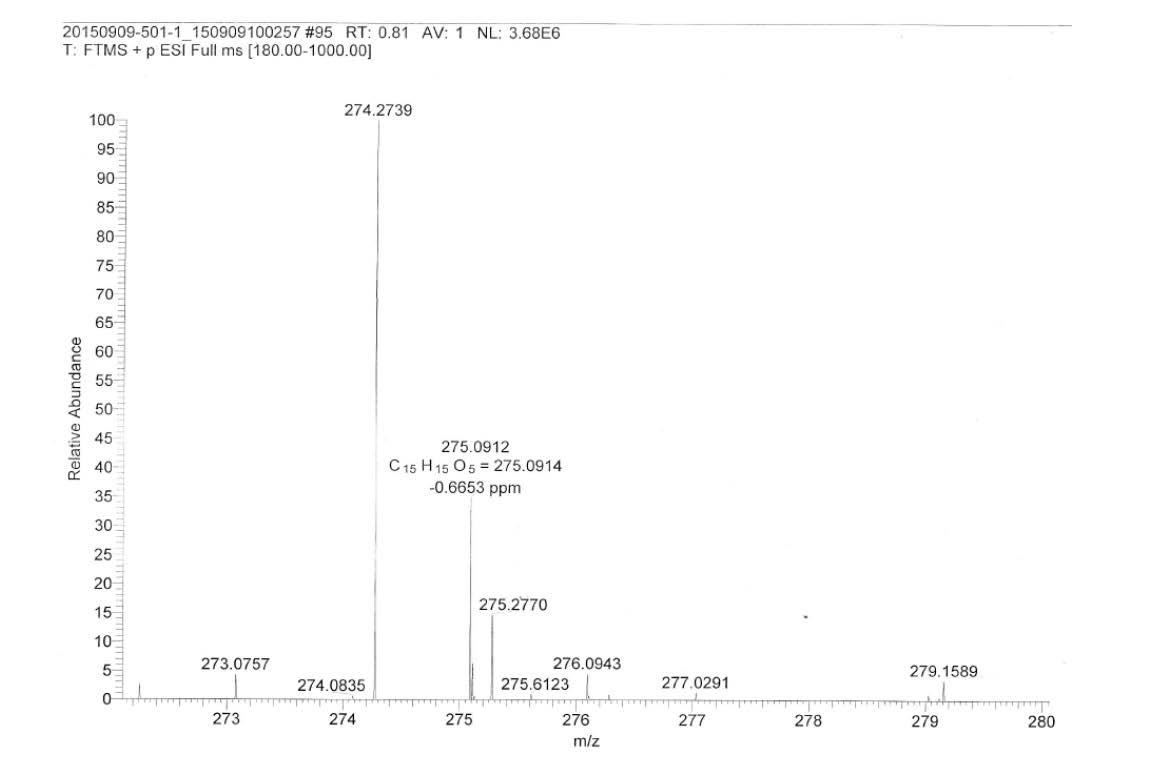


**Figure S7.** HRESIMS spectrum of compound **2**.


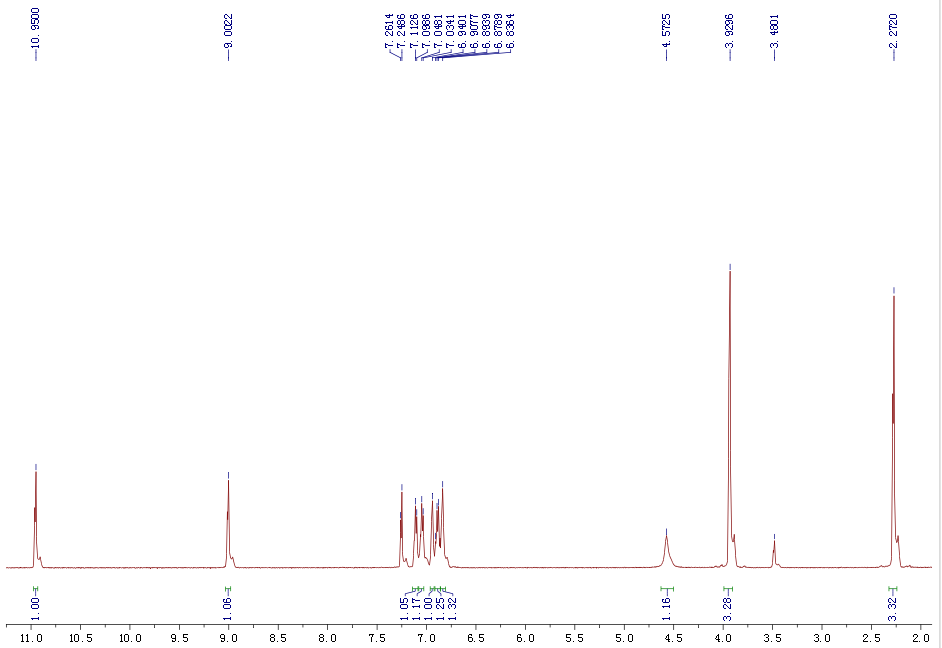


**Figure S8.**^1^H NMR (500 MHz, CDCl_3_) of compound **2**.


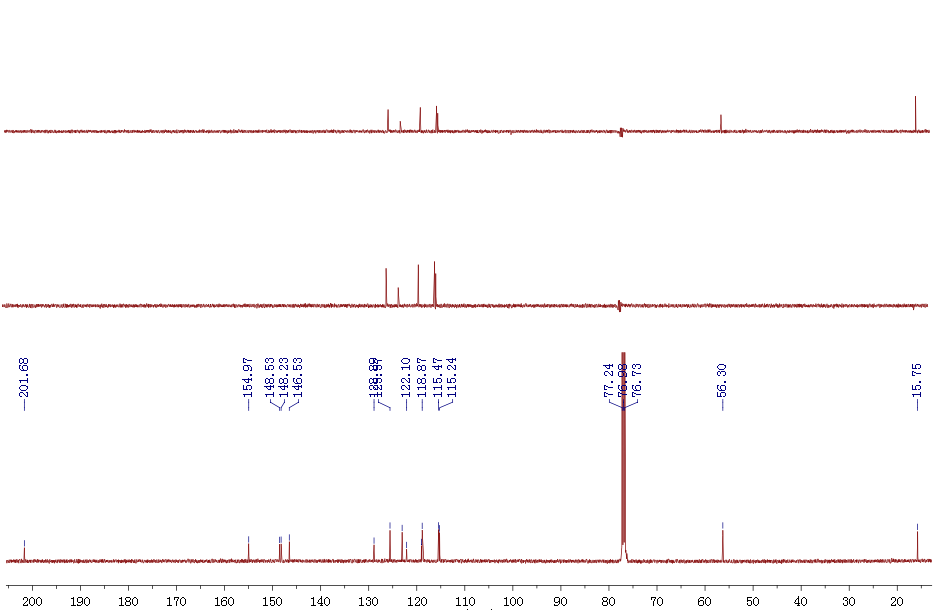


**Figure S9.**^13^C NMR and DEPT (125 MHz, CDCl_3_) of compound **2**.


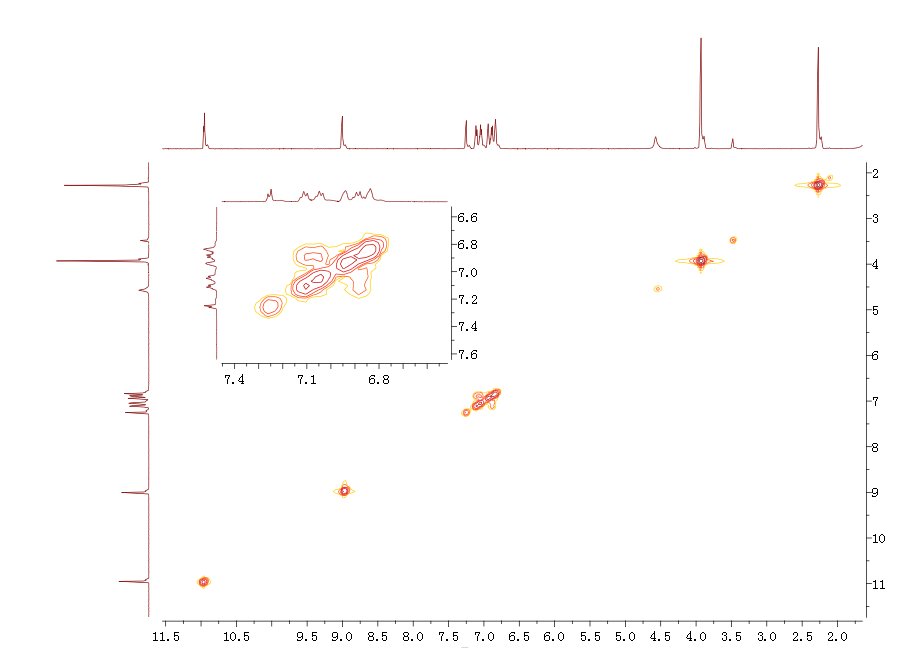


**Figure S10.** COSY spectrum of compound **2**.


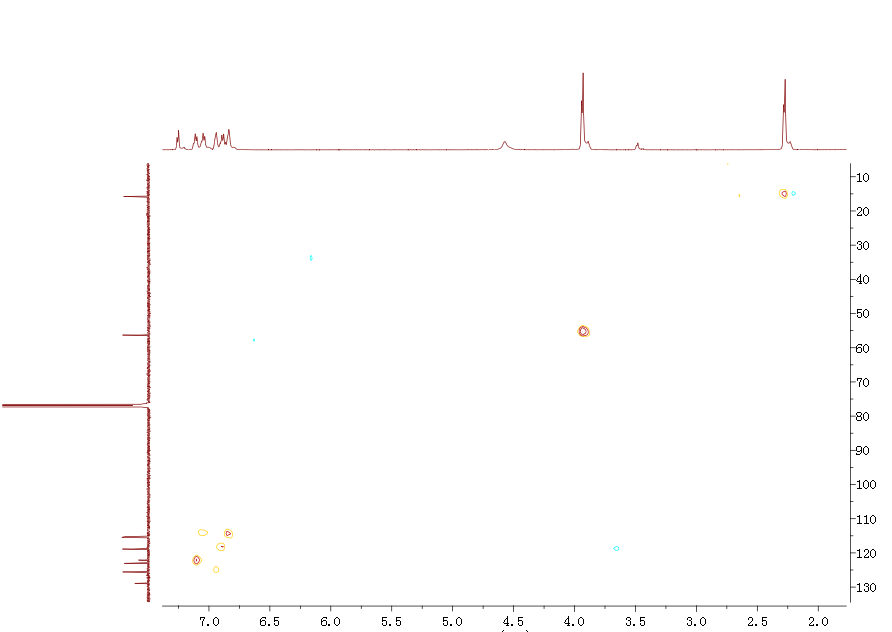


**Figure S11.** HSQC spectrum of compound **2**.


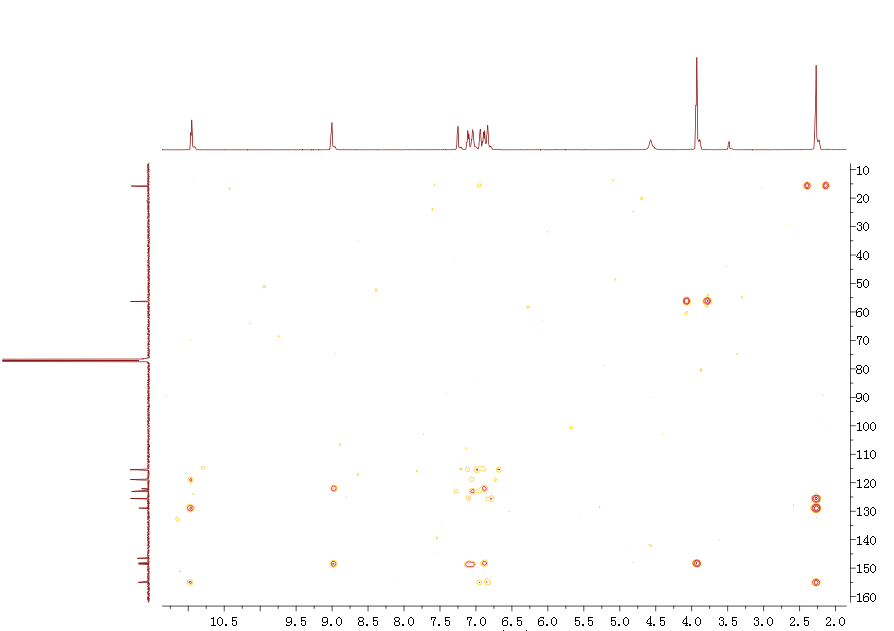


**Figure S12.** HMBC spectrum of compound **2**.


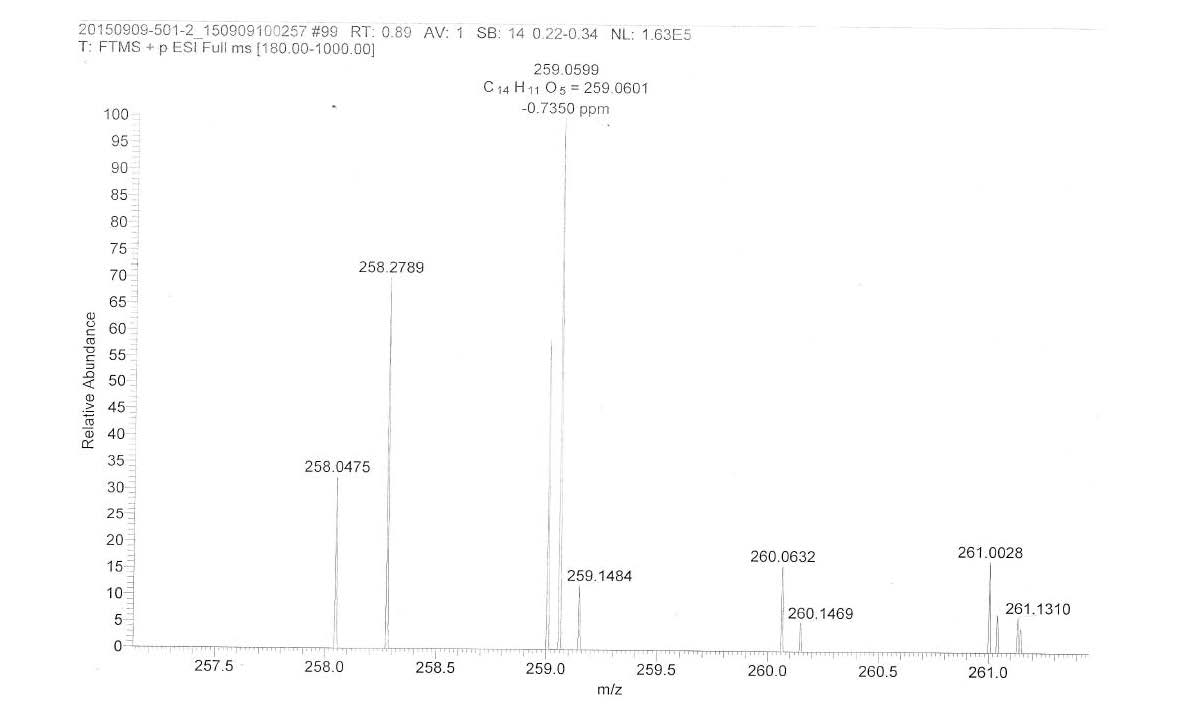


**Figure S13.** HRESIMS spectrum of compound **3**.


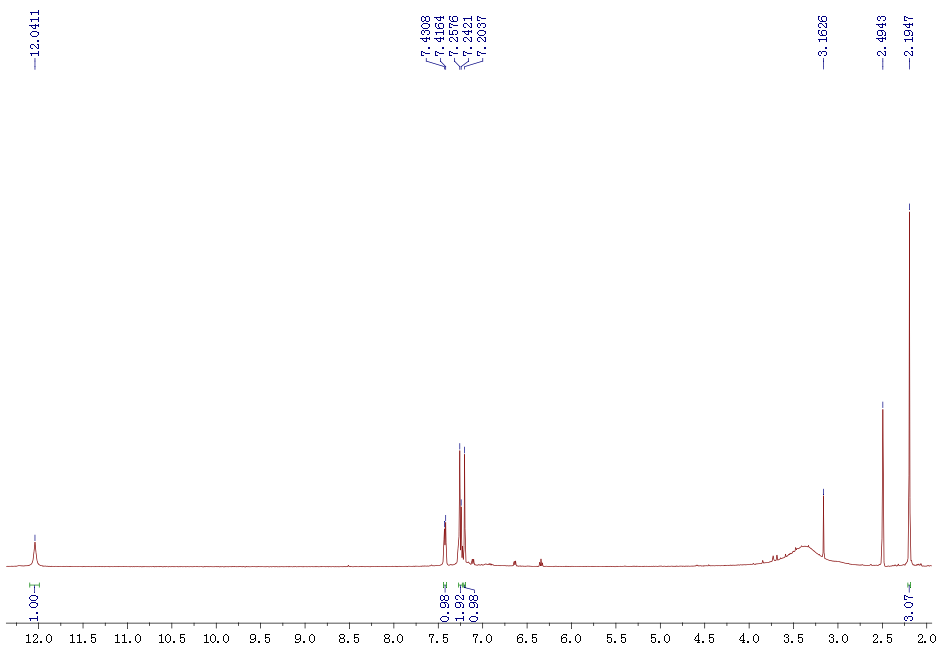


**Figure S14.**^1^H NMR (500 MHz, DMSO-*d*_6_) of compound **3**.


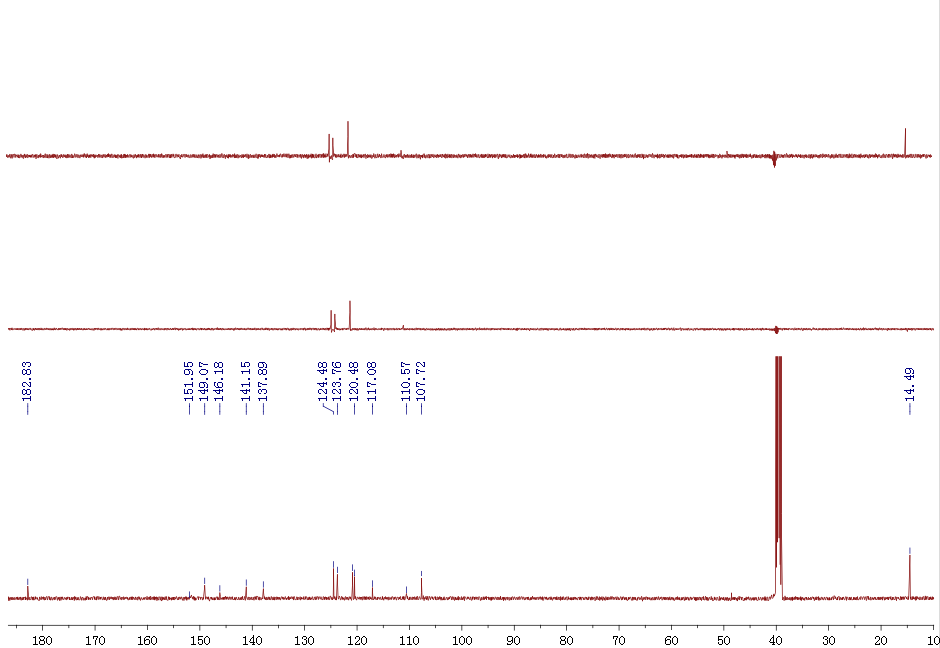


**Figure S15.**^13^C NMR and DEPT (125 MHz, DMSO-*d*_6_) of compound **3**.


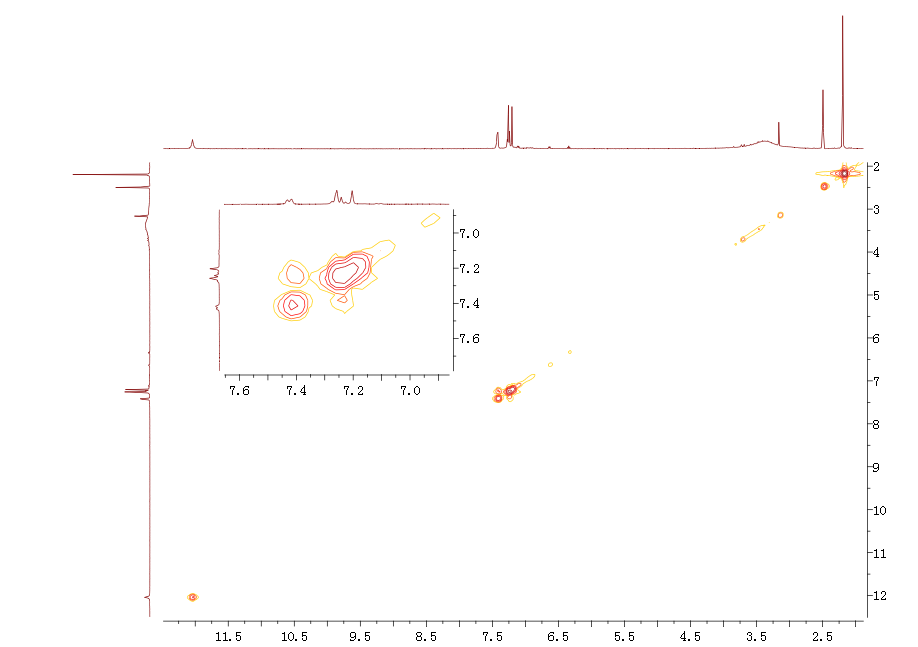


**Figure S16.** COSY spectrum of compound **3**.


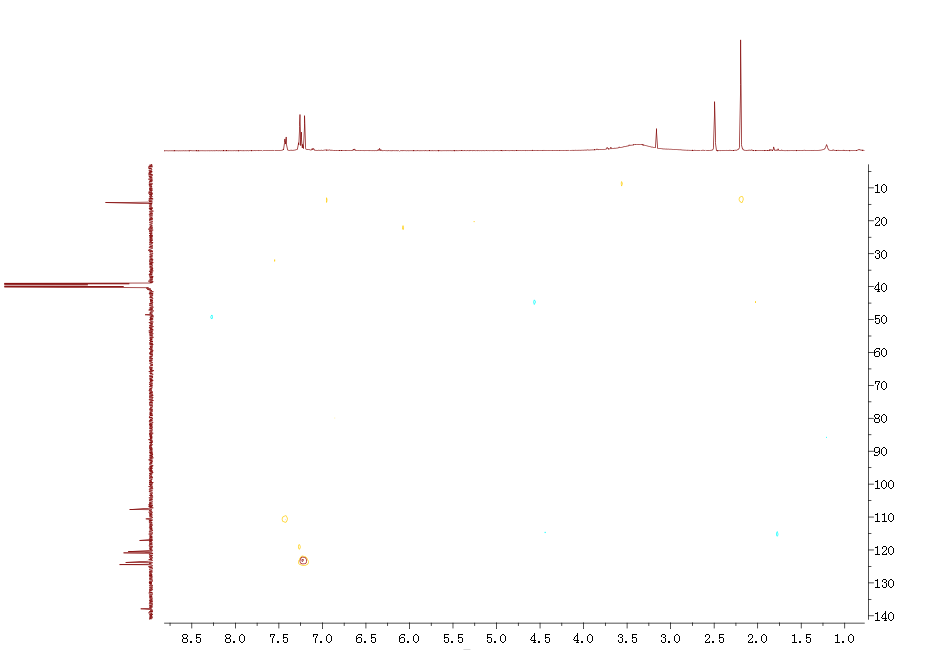


**Figure S17.** HSQC spectrum of compound **3**.


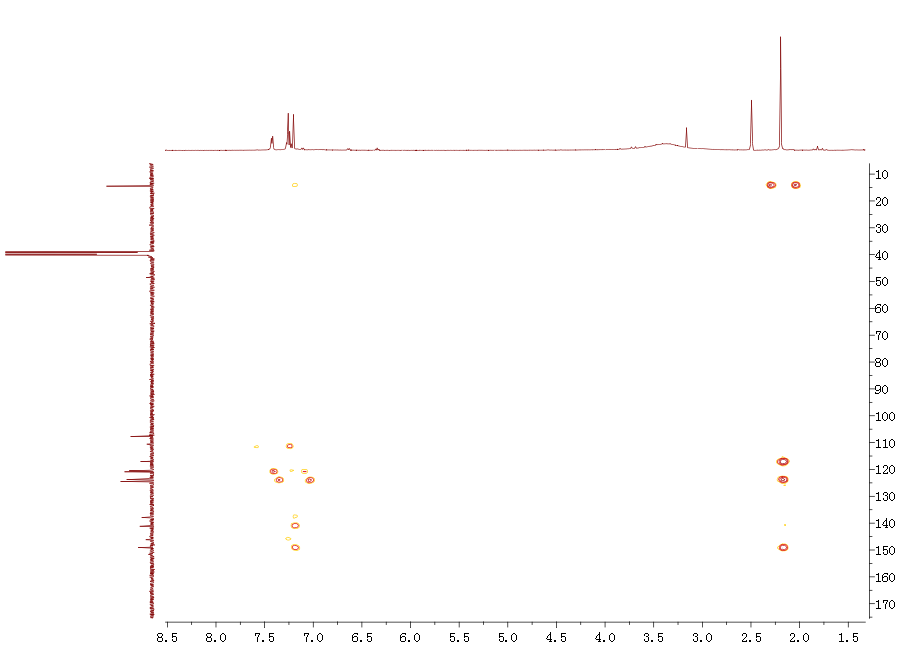


**Figure S18.** HMBC spectrum of compound **3**.


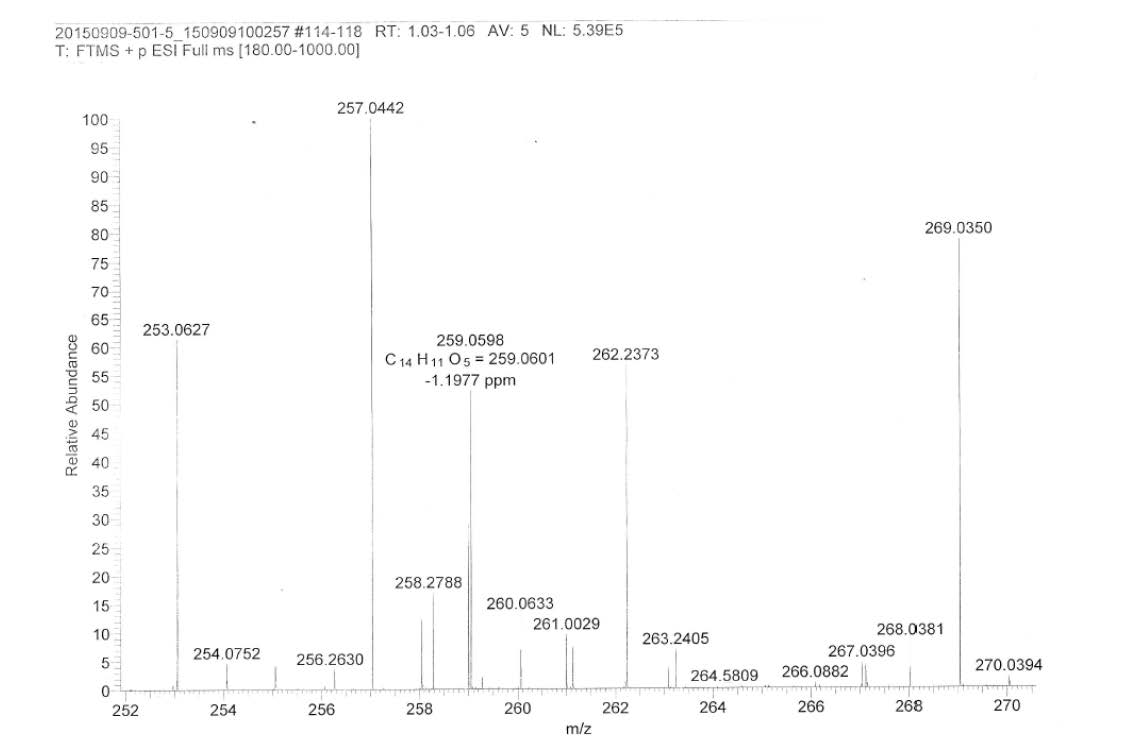


**Figure S19.** HRESIMS spectrum of compound **4**.


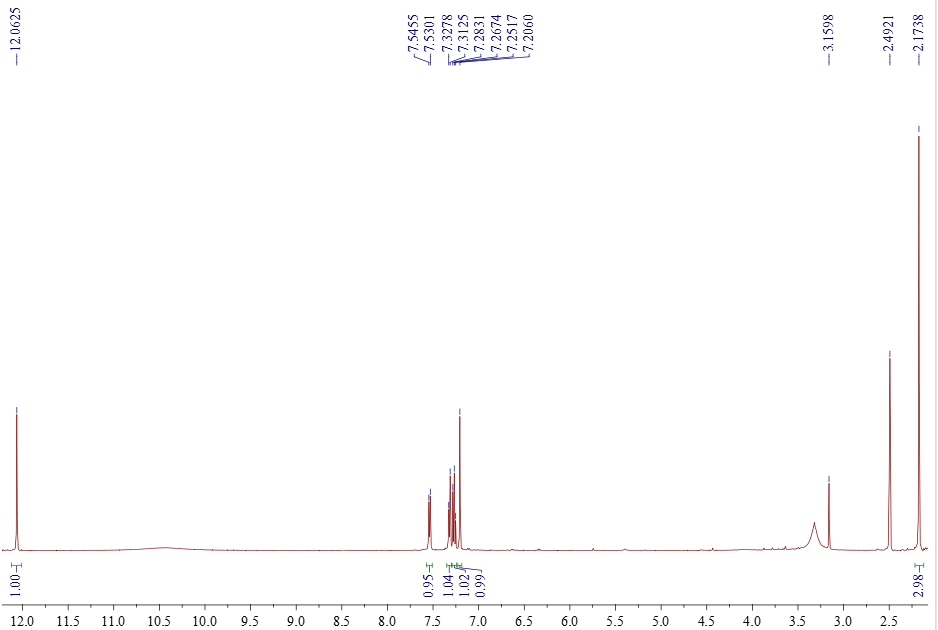


**Figure S20.**^1^H NMR (500 MHz, DMSO-*d*_6_) of compound **4**.


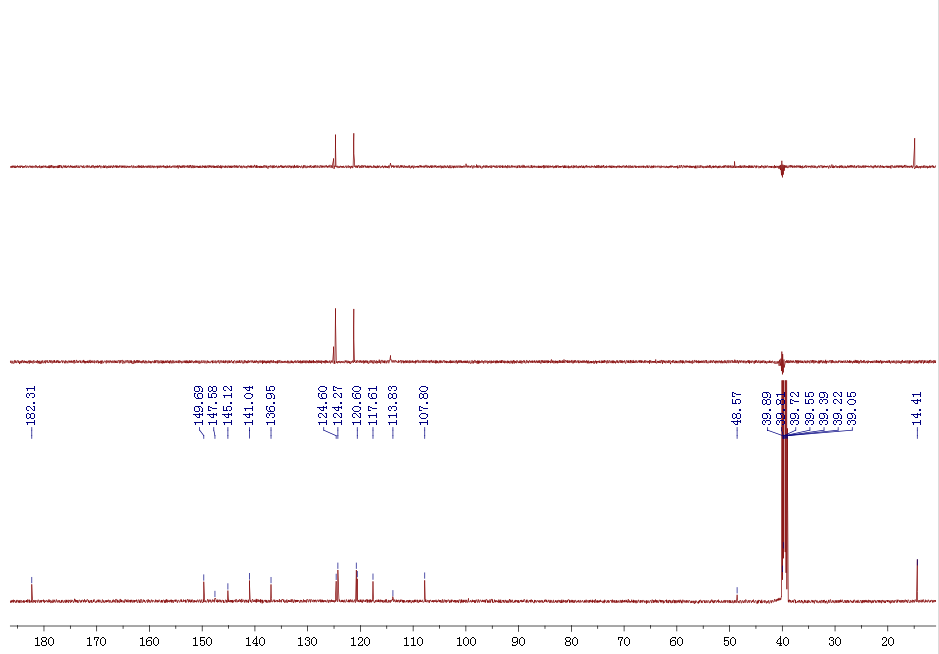


**Figure S21.**^13^C NMR and DEPT (125 MHz, DMSO-*d*_6_) of compound **4**.


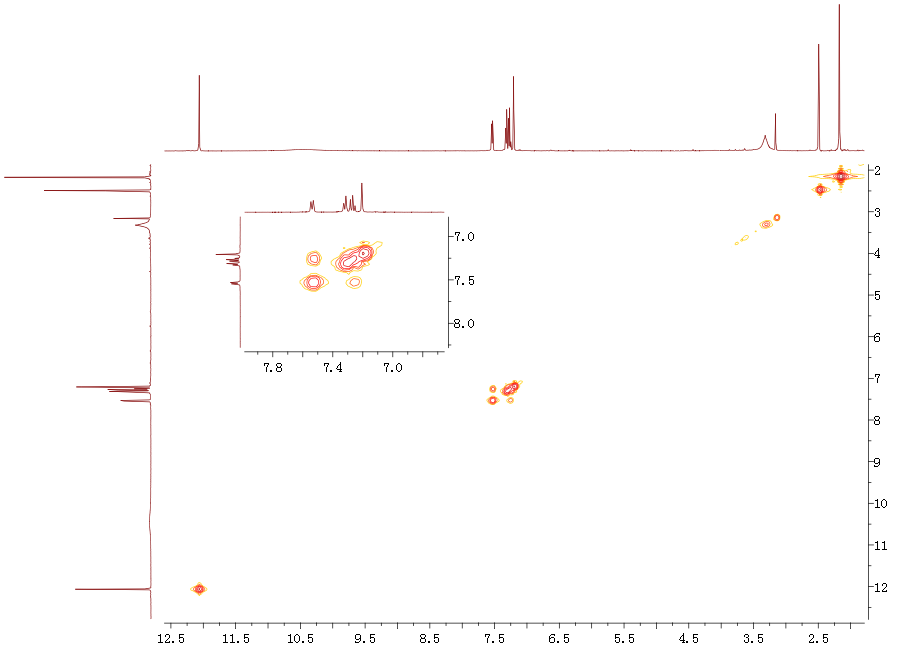


**Figure S22.** COSY spectrum of compound **4**.


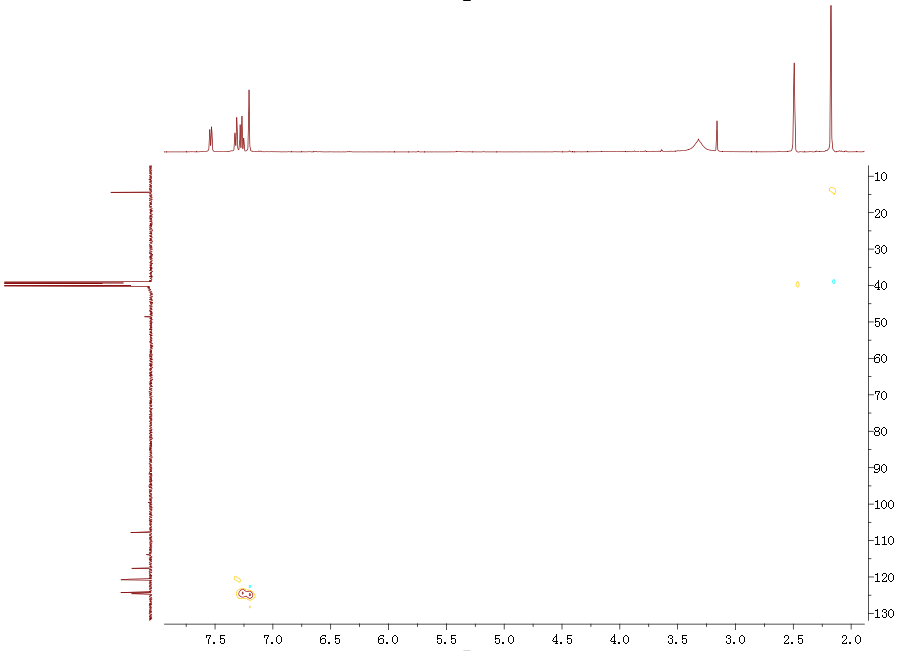


**Figure S23.** HSQC spectrum of compound **4**.


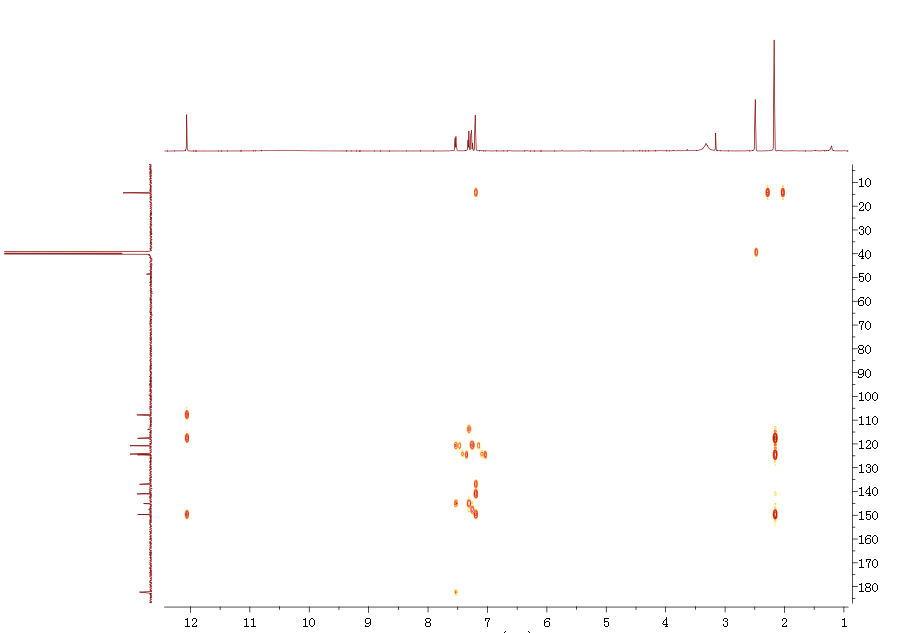


**Figure S24.** HMBC spectrum of compound **4**.
